# CHCHD4 Regulates Intracellular Oxygenation and Perinuclear Distribution of Mitochondria

**DOI:** 10.3389/fonc.2017.00071

**Published:** 2017-04-27

**Authors:** Luke W. Thomas, Oliver Staples, Mark Turmaine, Margaret Ashcroft

**Affiliations:** ^1^Department of Medicine, University of Cambridge, Cambridge Biomedical Campus, Cambridge, UK; ^2^Centre for Cell Signalling and Molecular Genetics, Division of Medicine, University College London, London, UK; ^3^Department of Cell and Developmental Biology, University College London, London, UK

**Keywords:** hypoxia, hypoxia-inducible factor-1α, mitochondria, mitochondrial localization, perinuclear, coiled-coil helix coiled-coil helix domain containing 4

## Abstract

Hypoxia is a characteristic of the tumor microenvironment and is known to contribute to tumor progression and treatment resistance. Hypoxia-inducible factor (HIF) dimeric transcription factors control the cellular response to reduced oxygenation by regulating the expression of genes involved in metabolic adaptation, cell motility, and survival. Alterations in mitochondrial metabolism are not only a downstream consequence of HIF-signaling but mitochondria reciprocally regulate HIF signaling through multiple means, including oxygen consumption, metabolic intermediates, and reactive oxygen species generation. CHCHD4 is a redox-sensitive mitochondrial protein, which we previously identified and showed to be a novel regulator of HIF and hypoxia responses in tumors. Elevated expression of *CHCHD4* in human tumors correlates with the hypoxia gene signature, disease progression, and poor patient survival. Here, we show that either long-term (72 h) exposure to hypoxia (1% O_2_) or elevated expression of CHCHD4 in tumor cells in normoxia leads to perinuclear accumulation of mitochondria, which is dependent on the expression of HIF-1α. Furthermore, we show that CHCHD4 is required for perinuclear localization of mitochondria and HIF activation in response to long-term hypoxia. Mutation of the functionally important highly conserved cysteines within the Cys-Pro-Cys motif of CHCHD4 or inhibition of complex IV activity (by sodium azide) redistributes mitochondria from the perinuclear region toward the periphery of the cell and blocks HIF activation. Finally, we show that CHCHD4-mediated perinuclear localization of mitochondria is associated with increased intracellular hypoxia within the perinuclear region and constitutive basal HIF activation in normoxia. Our study demonstrates that the intracellular distribution of the mitochondrial network is an important feature of the cellular response to hypoxia, contributing to hypoxic signaling *via* HIF activation and regulated by way of the cross talk between CHCHD4 and HIF-1α.

## Introduction

Low oxygenation (hypoxia) is a key feature of most human tumors and is associated with tumor progression and treatment resistance. Hypoxia activates a transcriptional program *via* the hypoxia-inducible factor (HIF) dimeric transcription factors, enabling tumor cells to metabolically adapt, survive, and metastasize. As the major sites of oxygen consumption within the cell, mitochondria control basal intracellular oxygenation (1) and cell metabolism (2). Mitochondrial reactive oxygen species (ROS) production and respiratory chain function regulate HIF activation (3). In addition, hypoxia and HIF transcriptional targets influence mitochondrial function (4–6). Of particular interest is the recent evidence showing that hypoxia leads to changes in mitochondrial morphology (7) and intracellular distribution (8) that are linked to reduced sensitivity to killing by cytotoxic agents (7) and HIF activation (8), respectively. However, the precise mitochondrial molecular mechanisms involved in tumor cell hypoxia responses that contribute to tumor progression and treatment resistance are not known.

Previously, we discovered that the redox-sensitive mitochondrial protein coiled-coil helix coiled-coil helix (CHCH) domain containing 4 (CHCHD4) controls basal cellular oxygen consumption rate (OCR), metabolic adaptive responses, HIF activation, and hypoxia signaling in tumor cells (9). We found that *CHCHD4* overexpression in human cancers correlates with the hypoxia gene signature and is associated with tumor progression and poor patient survival. CHCHD4 (known as Mia40 in yeast) is a key component of the disulfide relay system (DRS) within the mitochondrial intermembrane space (IMS) (9). As an oxidoreductase and part of the DRS, CHCHD4 functions to import and introduce disulfide bonds into proteins containing highly evolutionarily conserved cysteine motifs [e.g., twin-(CX_3_C) or twin-(CX_9_C) motif] that are essential for mitochondrial function (9–12). In doing so, electrons are generated and transferred *via* the DRS to cytochrome c and complex IV (the molecular oxygen accepting complex) of the respiratory chain (13). CHCHD4 mitochondrial localization and import function are dependent on the highly evolutionarily conserved cysteines within the CHCH domain and redox-sensitive Cys-Pro-Cys (CPC) motif (9, 10). CHCHD4 substrates are involved in various aspects of mitochondrial biology, including the assembly and activity of respiratory chain complexes, modeling of the cristae, lipid biosynthesis, and protein import into the matrix [reviewed in Ref. (12)].

In this study, we evaluate the contribution of CHCHD4 mitochondrial function to HIF signaling in tumor cells. We show that increased expression of CHCHD4 in tumor cells leads to intracellular hypoxia and constitutive activation of HIF. We show that either long-term exposure of cells to hypoxia (72 h) or increased expression of CHCHD4 in tumor cells leads to (i) the redistribution of the mitochondria to the perinuclear region of the cell, (ii) HIF-1α stabilization, and (iii) the upregulation of HIF targets. Hypoxic perinuclear localization of mitochondria is dependent on CHCHD4. CHCHD4-mediated perinuclear localization of mitochondria in normoxia correlates with elevated intracellular hypoxia and requires the highly conserved cysteines within the CPC motif. Finally, we show that both hypoxic and CHCHD4-mediated perinuclear accumulation of mitochondria is dependent on HIF-1α expression. Our data highlight for the first time the importance of CHCHD4 in regulating mitochondrial subcellular localization, intracellular oxygenation, and HIF activation in tumor cells.

## Results

### Hypoxia Induces Perinuclear Localization of Mitochondria

**Figure 1:**
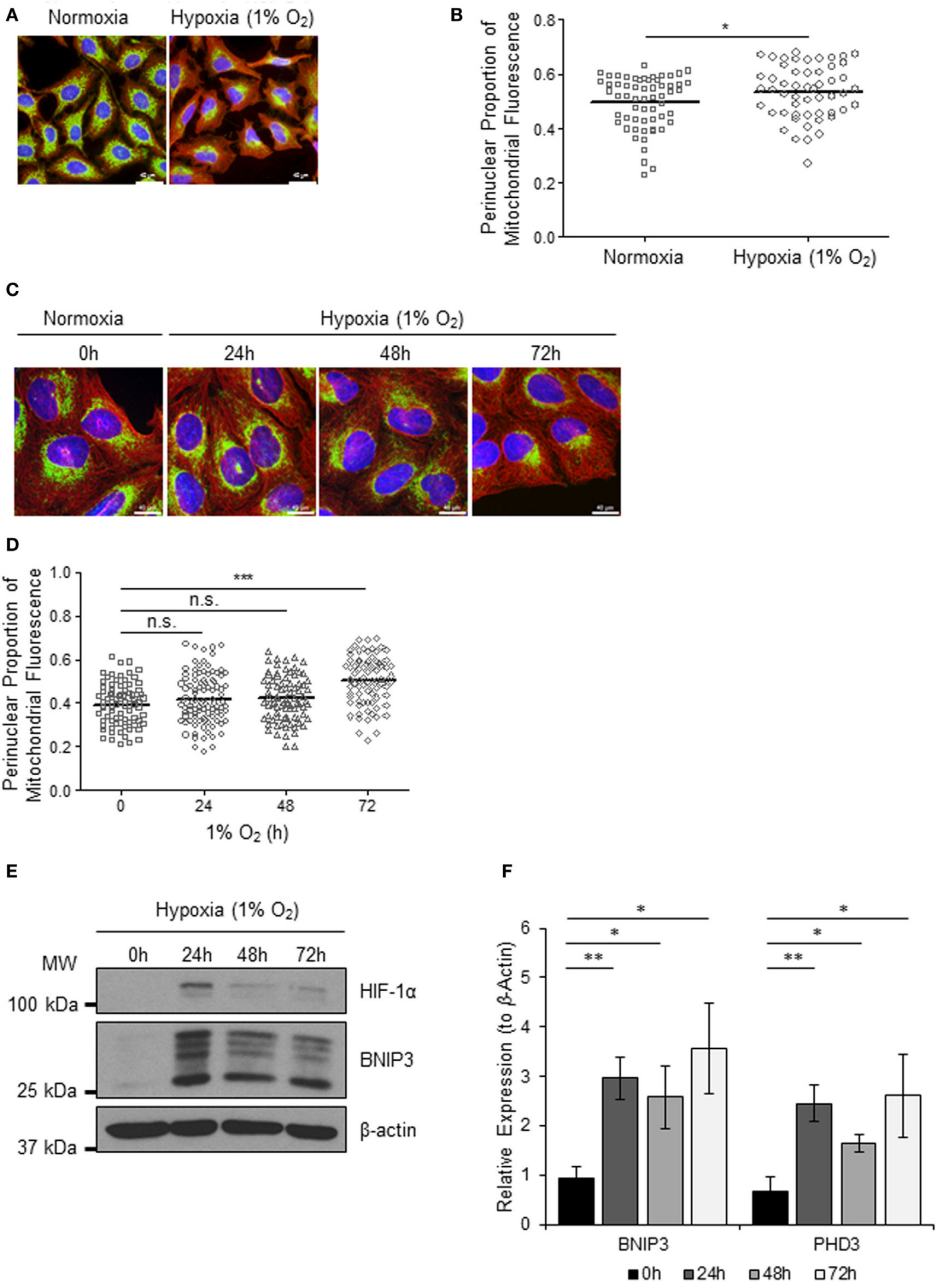
**Hypoxia induces perinuclear localization of mitochondria**. **(A)** Images of immunostaining analyses of HeLa cells incubated in normoxia or hypoxia (1% O_2_) for 72 h. Cells were immunostained for COXIV (mitochondria, green) and α-tubulin (cytoskeleton, red). Nuclei were stained with DAPI (blue). Scale bar = 40 µm. **(B)** Graph shows the perinuclear proportion of mitochondrial fluorescence per cell, analyzed from cells described in **(A)** (10 fields of view per condition). *p < 0.05. **(C)** Images of U2OS cells incubated in normoxia or hypoxia (1% O_2_) for the indicated times. Cells were immunostained as in **(A)**. Scale bar = 40 µm. **(D)** Graph shows the perinuclear proportion of mitochondrial fluorescence per cell, analyzed from cells described in **(C)** (10 fields of view per condition). *n* = 3; n.s., not significant; ***p < 0.001. **(E)** Western blots show hypoxia-inducible factor (HIF)-1α and BNIP3 protein levels in U2OS cells described in **(C)**. β-actin was used as a load control. **(F)** Graph shows expression of *BNIP3* and *PHD3* analyzed by QPCR using total RNA isolated from U2OS cells incubated in normoxia (0 h) or hypoxia (1% O_2_) for 24, 48, and 72 h. *p < 0.05, **p < 0.01.

Mitochondria respond to exogenous hypoxia in numerous ways including regulating oxygen consumption and respiratory chain efficiency (5) and controlling metabolic signaling pathways (14) in order to maintain cellular energy homeostasis and cell viability when oxygen is limiting. In addition, other mitochondrial parameters such as mitochondrial dynamics (7, 15) and subcellular localization are also known to be influenced by hypoxia (8, 16) *via* mechanisms that are not yet understood. To explore the cross talk between mitochondria and the hypoxia (HIF) response, we first set out to investigate the effect of hypoxia on mitochondrial morphology and intracellular distribution of the mitochondrial network over a prolonged time course. We found that 72 h of exposure to 1% O_2_, but not 24 or 48 h, led to a visible redistribution of the mitochondrial network from the periphery of the cell to the perinuclear region, in both HeLa (Figures 1A,B) and in human U2OS osteosarcoma (U2OS-HRE-luc) cells (Figures 1C,D). U2OS-HRE-luc cells were generated by us previously (17). Digital analysis of the distribution of mitochondrial fluorescence across cytoplasmic segments confirmed a small but significant redistribution of the mitochondria to the perinucleus in HeLa (Figure 1B) and U2OS (Figure 1D) cells. HIF-1α protein stabilization over this hypoxia time course was strongest at 24 h in U2OS cells and declined by 48 h, but HIF was still transcriptionally active at 72 h, as indicated by sustained upregulated expression of the HIF targets *BNIP3* (Figures 1E,F) and *PHD3* (Figure 1F).

### CHCHD4 Regulates Perinuclear Localization of Mitochondria and Mitochondrial Morphology

**Figure 2:**
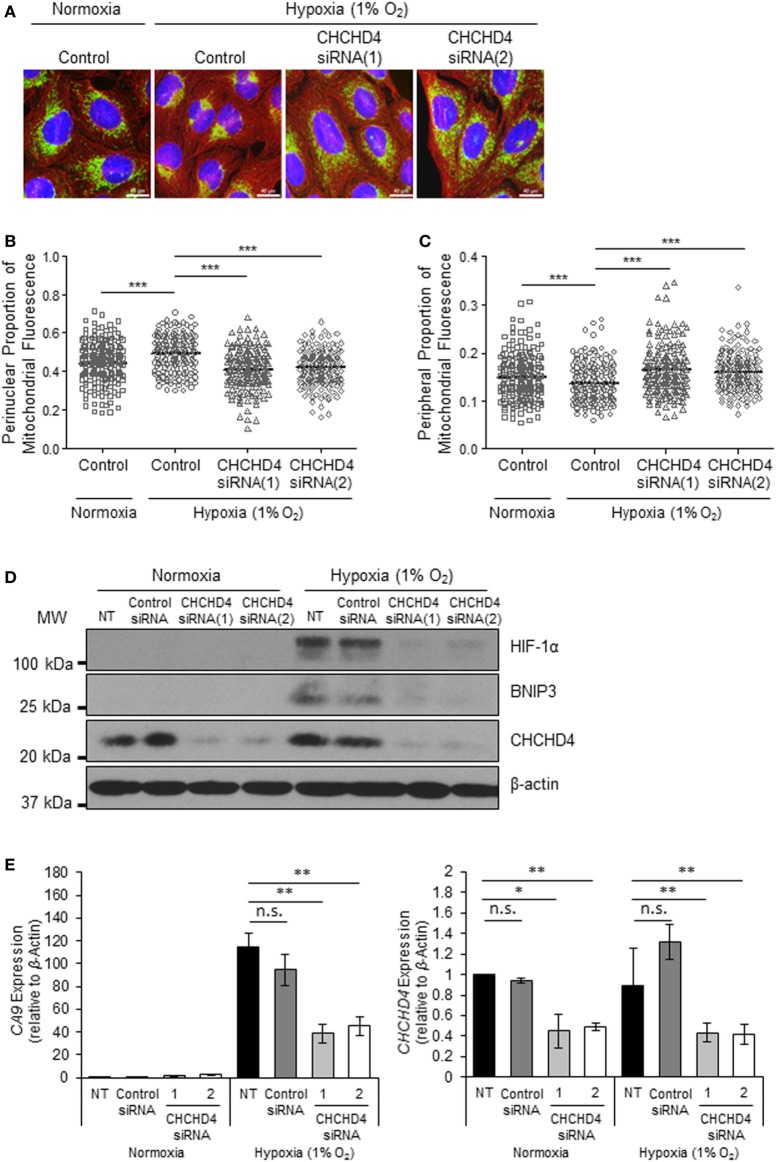
**CHCHD4 is required for perinuclear localization of mitochondria in hypoxia**. **(A)** Images of immunostaining analyses of U2OS cells incubated in normoxia or hypoxia (1% O_2_) for 72 h. Cells were untreated (control) or transfected with siRNAs targeting *CHCHD4* [siRNA(1) and (2)]. Cells were immunostained for COXIV (mitochondria, green) and α-tubulin (cytoskeleton, red). Nuclei were stained with DAPI (blue). Scale bar = 40 µm. **(B,C)** Graphs show the perinuclear **(B)** or peripheral **(C)** proportion of mitochondrial fluorescence per cell, analyzed from cells described in **(A)** (10 fields of view per condition). *n* = 3; ***p < 0.001. **(D)** Western blots show hypoxia-inducible factor (HIF)-1α, BNIP3, and CHCHD4 protein levels in U2OS cells described in **(A)**. β-actin was used as a load control. **(E)** Graphs show expression of *CA9* and *CHCHD4* analyzed by QPCR using total RNA isolated from U2OS cells incubated in normoxia or hypoxia (1% O_2_) for 72 h. Cells were untreated (NT), treated with non-silencing control (control siRNA) or siRNAs targeting *CHCHD4* [siRNA(1) and (2)]. Mean ± SD. *n* = 2. n.s., not significant; **p < 0.01.

Recently, we identified the mitochondrial protein CHCHD4 as a critical regulator of HIF-1α stabilization and signaling in hypoxia through its effects on mitochondrial function (9). For this reason, we next investigated whether CHCHD4 was required for the hypoxic redistribution of mitochondria to the perinucleus. Depletion of CHCHD4 using two independent siRNAs significantly reduced the perinuclear accumulation of the mitochondrial network in cells incubated for 72 h at 1% O_2_ (Figures 2A,B) resulting in a more peripheral distribution of mitochondria (Figure 2C). Consistent with our previous study (9), CHCHD4 knockdown also blocked the hypoxic stabilization of HIF-1α protein and the transcriptional upregulation of HIF targets, including BNIP3 (Figure 2D) and *CA9* (Figure 2E). These data suggest that CHCHD4 expression is required for the perinuclear redistribution of the mitochondria in response to hypoxia and also indicate that there may be a HIF-dependent aspect to this phenomenon.

**Figure 3:**
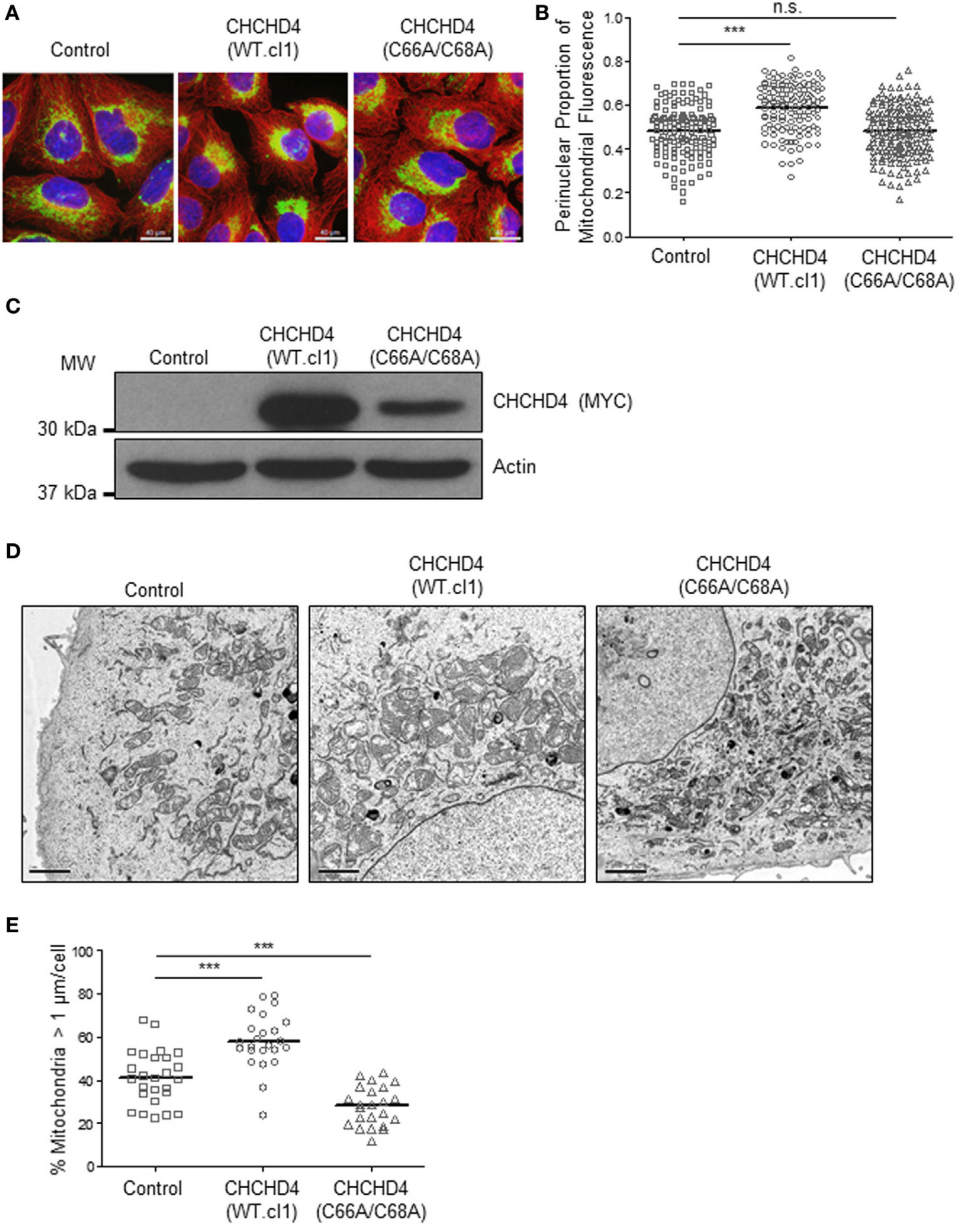
**CHCHD4 regulates perinuclear localization of mitochondria and mitochondrial morphology**. **(A)** Images of immunostaining analysis of U2OS cells (control), CHCHD4 wild-type (WT)-expressing (WT.cl1) cells, or CHCHD4 (C66A/C68A)-expressing cells. Cells were immunostained for COXIV (mitochondria, green) and α-tubulin (cytoskeleton, red). Nuclei were stained with DAPI (blue). Scale bar = 40 µm. **(B)** Graph shows the perinuclear proportion of mitochondrial fluorescence per cell, analyzed from cells described in **(A)** (10 fields of view per condition). *n* = 3; n.s., not significant, ***p < 0.001. **(C)** Western blots show exogenously expressed CHCHD4 protein levels (immunoblotted for the myc-epitope tag) in cells described in **(A)**. β-actin was used as a load control. **(D)** Electron microscope images of cells described in **(A)**. Scale bar = 2 µm. **(E)** Graph shows % mitochondria >1 µm/cell from images described in **(D)**. *n* = 25 cells, ***p < 0.001.

Previously, we discovered that CHCHD4 regulates HIF-1α protein stability and HIF activity in both loss and gain of function systems (9). Therefore, we next investigated whether the mitochondrial network was distributed differently in independent clonal U2OS cell lines stably expressing wild-type (WT) CHCHD4 [CHCHD4 (WT)-expressing cells, clones WT.cl1 or WT.cl3]. Under normoxic conditions, the localization of the mitochondrial network in CHCHD4 (WT)-expressing cells was significantly more perinuclear compared with control cells (Figures 3A–C). However, stable expression of a mutant form of CHCHD4, in which the highly conserved cysteines of the CPC motif have been changed to alanines (C66A/C68A) (9), did not lead to perinuclear accumulation of the mitochondrial network (Figures 3A–C). We (9) and others (10) have previously shown that the highly conserved cysteines within CHCHD4 are required for its mitochondrial localization (9) and import function (10). These data suggest that the effects of CHCHD4 on mitochondrial subcellular distribution are dependent on its mitochondrial localization and import function. Interestingly, in both primary human and yeast cells harboring mutations in the sulfhydryl oxidase GFER (Erv1 in yeast), which is essential for the redox activity of CHCHD4 (Mia40 in yeast), the mitochondria show significant changes in both their distribution and morphology (18, 19).

Changes in mitochondrial dynamics (fission and fusion) are also known to underlie changes in the distribution of the mitochondrial network and have been detected under both short- (20) and long-term exposure to hypoxia (7). Using electron microscopy, we confirmed the perinuclear localization of the mitochondria in CHCHD4 (WT)-expressing cells and observed larger mitochondria (>1 μm in diameter) compared with control cells, suggesting that elevated CHCHD4 expression causes increased fusion of the mitochondrial network (Figures 3D,E). Consistently, we observed reduced expression of the mitochondrial fission protein dynamin-related protein 1 (DRP-1) (21) in CHCHD4 (WT)-expressing cells (data not shown), which is a protein previously shown to be involved in regulating perinuclear localization of mitochondria (15). Conversely, in CHCHD4 (C66A/C68A)-expressing cells, we observed a significantly reduced number of larger mitochondria (>1 μm in diameter) compared to control cells, suggesting a more fragmented phenotype, similar to that described in GFER mutant cells (18).

### Elevated CHCHD4 Expression Causes Localized Intracellular Hypoxia within the Perinuclear Region and Constitutive HIF Activation

**Figure 4:**
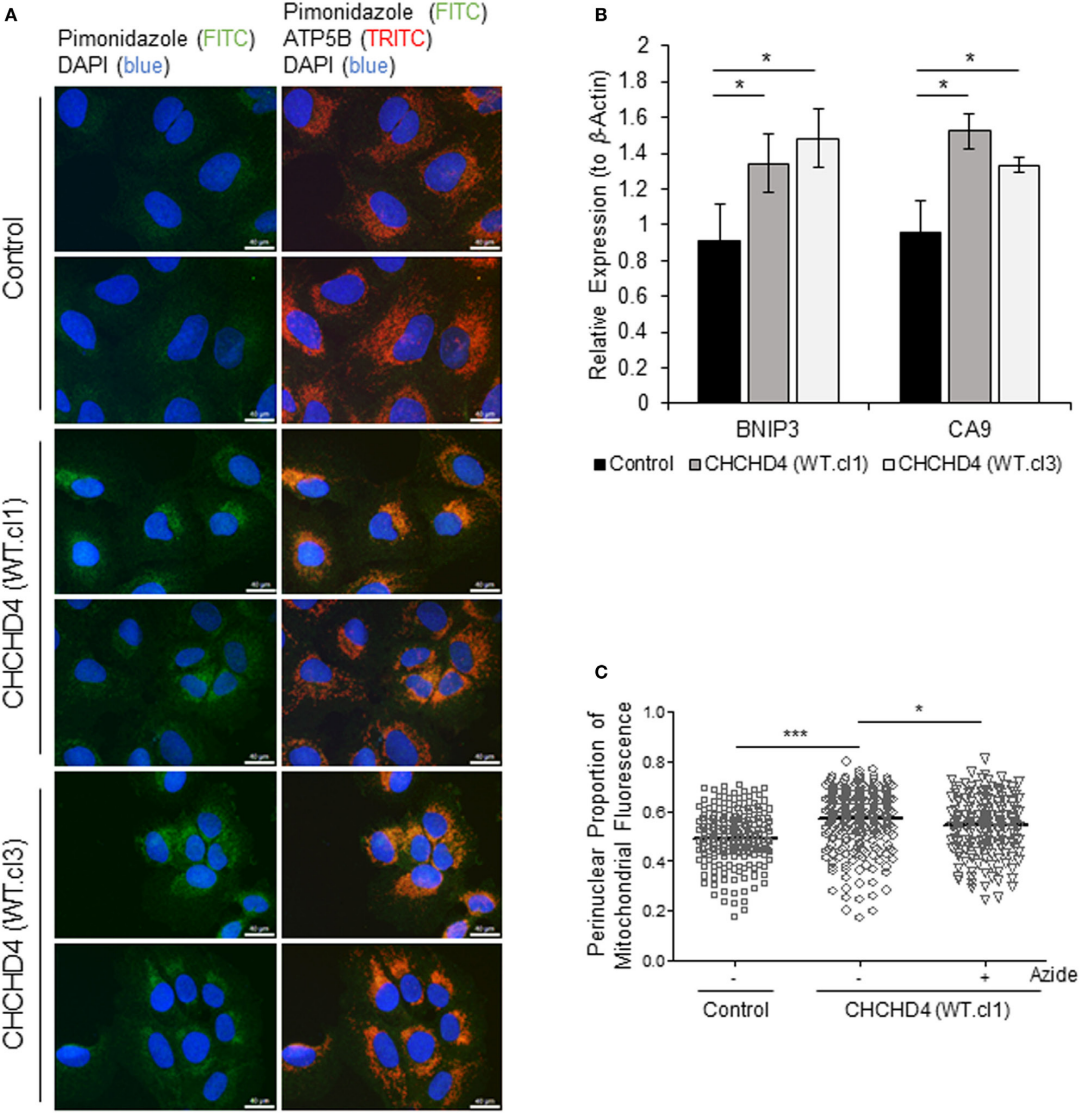
**Elevated CHCHD4 expression causes localized intracellular hypoxia within the perinuclear region and constitutive hypoxia-inducible factor activation**. **(A)** Images of immunostaining of U2OS cells (control) and CHCHD4 wild-type (WT)-expressing cells (clones WT.cl1 and WT.cl3) incubated with pimonidazole for 2 h. Cells were immunostained for pimonidazole (green) and ATP5B (mitochondria, red). Nuclei stained with DAPI (blue). Scale bar = 40 µm. **(B)** Graph shows expression of *BNIP3* and *CA9* analyzed by QPCR using total RNA isolated from cells described in **(A)**. Mean ± SD. *p < 0.05. **(C)** Graph shows the perinuclear proportion of mitochondrial fluorescence per cell, analyzed from cells described in **(A)**, after treatment without (−) or with (+) sodium azide (azide, 5 mM) for 16 h (10 fields of view per condition). *n* = 3; ***p < 0.001, *p < 0.05.

It has been shown that the perinuclear accumulation of the mitochondria after short-term exposure to hypoxia is required for the efficient delivery of mitochondrial ROS to the (nuclear) promoters of key HIF target genes such as *VEGF*. We (9) and others (22) have proposed that mitochondria are responsible for the generation of intracellular oxygen gradients and are associated with local regions of reduced oxygenation within the cell. Furthermore, previously, we have discovered that elevated expression of CHCHD4 in tumor cells leads to increased OCR (9). Therefore, we hypothesized that CHCHD4-mediated perinuclear localization of mitochondria may lead to increased intracellular hypoxia within the perinuclear region of the cell. To investigate this, we used the hypoxia-marker pimonidazole (23) and immunofluorescence microscopy. Notably, pimonidazole adduct formation is oxygen-dependent and occurs independently of changes in pyridine nucleotide redox state (i.e., NADP/H) (24). As anticipated, in CHCHD4 (WT)-expressing cells, we observed increased pimonidazole staining co-localized with mitochondria within the perinuclear region of the cell in normoxia (Figure 4A). We confirmed the elevated perinuclear hypoxia in CHCHD4 (WT)-expressing cells using a second fluorescent hypoxia marker, HypoxiTRAK, by live cell imaging (data not shown). Concurrently, we observed significantly upregulated expression of the HIF-target gene transcripts, *CA9* and *BNIP3* in the CHCHD4 (WT)-expressing U2OS cells compared to control cells (Figure 4B), indicating above basal HIF-signaling in these cells. Interestingly, we have discovered previously that CHCHD4-mediated enhanced HIF-1α protein stability and HIF activation is sensitive to inhibition by the complex IV inhibitor, sodium azide (9). Therefore, next, we assessed whether CHCHD4-mediated perinuclear localization of mitochondria was also affected by sodium azide treatment. Indeed, we found that sodium azide inhibited complex IV activity (data not shown) and significantly reduced the perinuclear localization of mitochondria (Figure 4C) and associated intracellular hypoxia (data not shown) in the CHCHD4 (WT)-expressing cells. Collectively our data suggest that CHCHD4-mediated perinuclear accumulation of mitochondria is capable of generating associated localized areas of intracellular hypoxia within the perinuclear region of the cell that are sufficient to lead to HIF transcriptional activation in normoxia. Furthermore, these effects are dependent on respiratory chain function (at least in part *via* complex IV).

### Perinuclear Accumulation of Mitochondria Is HIF-1α-Dependent

**Figure 5:**
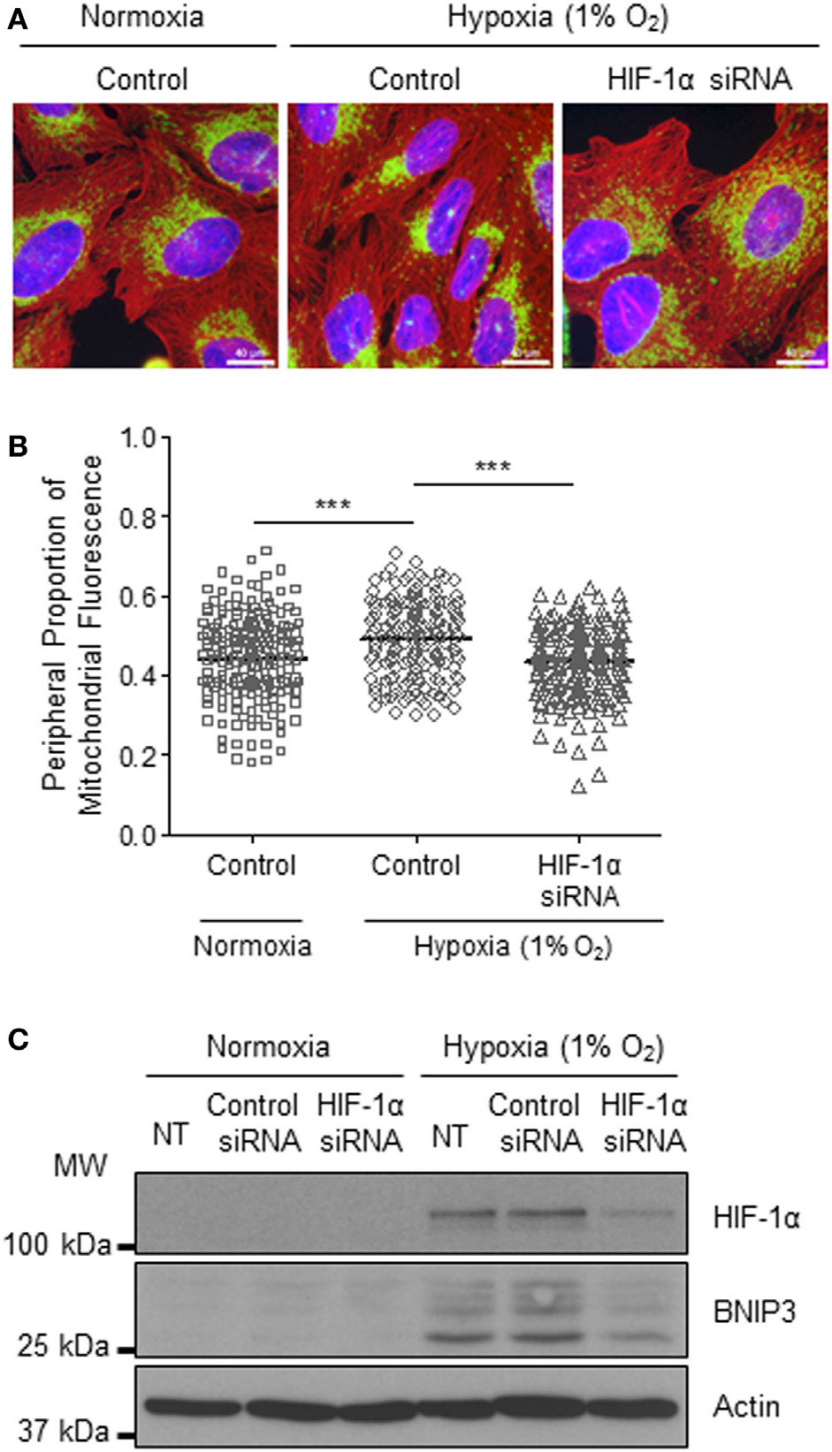
**Hypoxia-inducible factor (HIF)-1α knockdown reverses hypoxia-induced perinuclear localization of mitochondria**. **(A)** Images of immunostaining of U2OS cells incubated in normoxia or hypoxia (1% O_2_) for 72 h. Cells were untreated (control) or transfected with siRNA targeting *HIF1A* (HIF-1α siRNA). Cells were immunostained for COXIV (mitochondria, green), and α-tubulin (cytoskeleton, red). Nuclei were stained with DAPI (blue). Scale bar = 40 µm. **(B)** Graph shows the perinuclear proportion of mitochondrial fluorescence per cell, analyzed from cells described in **(A)** (10 fields of view per condition). *n* = 3; ***p < 0.001. **(C)** Western blots show HIF-1α and BNIP3 protein levels in U2OS cells untreated (NT), or transfected with a non-silencing control (control siRNA) or siRNA targeting *HIF1A* (HIF-1α siRNA). Cells were incubated in normoxia or hypoxia (1% O_2_) for 72 h. β-actin was used as a load control.

**Figure 6:**
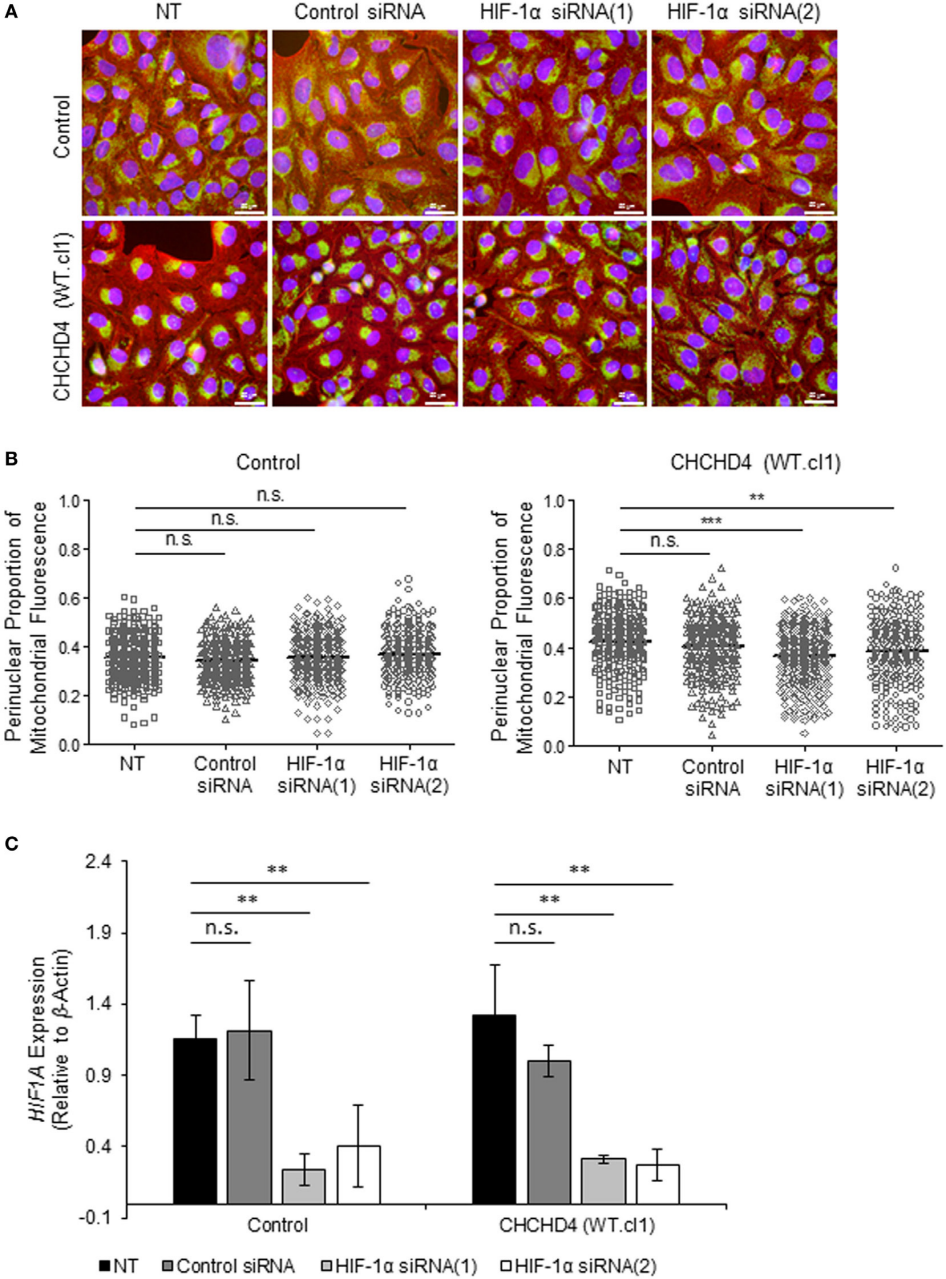
**Hypoxia-inducible factor (HIF)-1α knockdown reverses CHCHD4-induced perinuclear localization of mitochondria**. **(A)** Images show control U2OS cells (control) and CHCHD4 (WT)-expressing cells (WT.cl1), which were untreated (NT) or transfected with siRNAs targeting *HIF1A* [HIF-1α siRNA (1) and (2)]. Cells were immunostained for COXIV (mitochondria, green) and α-tubulin (cytoskeleton, red). Nuclei were stained with DAPI (blue). Scale bar = 50 µm. **(B)** Graphs show the perinuclear proportion of mitochondrial fluorescence per cell, analyzed from cells described in **(A)** [10 fields of view for each control cells (left graph), and CHCHD4 WT-expressing cells (WT.cl1, right graph)]. *n* = 3; n.s., not significant; ***p < 0.001. **(C)** Graph shows expression of *HIF1A* analyzed by QPCR using total RNA isolated from cells described in **(A)**. Mean ± SD. *n* = 3. n.s., not significant; **p < 0.01.

The HIF transcription factor family constitute the major molecular regulators of the cellular response to hypoxia (25, 26) and are known to upregulate the expression of proteins involved in mitochondrial morphology and distribution (16). Therefore, next, we investigated whether perinuclear redistribution of mitochondria in response to prolonged hypoxia was dependent on HIF-1α expression. Interestingly, we found that siRNA-mediated depletion of HIF-1α blocked the perinuclear accumulation of mitochondria in U2OS cells incubated for 72 h in 1% O_2_ (Figures 5A–C). HIF-1α knockdown also blocked hypoxic upregulation of the HIF transcriptional target BNIP3 (Figure 5C). Furthermore, depletion of HIF-1α using two independent siRNAs reversed the perinuclear accumulation of mitochondria in CHCHD4 (WT)-expressing cells (Figures 6A,B). However, we observed no significant effect of HIF-1α siRNA knockdown on the distribution of mitochondria in control cells (Figures 6A,B), despite significant knockdown of *HIF1A* expression (Figure 6C).

Our data highlight CHCHD4 as a critical regulator of mitochondrial distribution and morphology. Perinuclear accumulation of mitochondria in response to hypoxia or elevated CHCHD4 is associated with increased intracellular hypoxia within the perinuclear region and elevated HIF-1α stabilization and HIF transcriptional activation. Taken together, our study indicates that CHCHD4 can control the distribution of the mitochondrial network in tumor cells through its role in regulating respiratory chain function, intracellular oxygenation, and HIF activation.

## Materials and Methods

### Cell Culture

Human U2OS osteosarcoma (U2OS-HRE-luc) cells have been described by us previously (17) and stably express a luciferase reporter construct under the control of a hypoxia response element, allowing us to monitor HIF/HRE activity alongside measuring mitochondrial endpoints. U2OS osteosarcoma (U2OS) cells were used to generate stable independent clonal cell lines (WT.cl1 and WT.cl3)-expressing CHCHD4.1 cDNA (CHCHD4 (WT)-expressing cells), CHCHD4-C66A/C668A cDNA (CHCHD4 (C66A/C68A)-expressing cells) or pcDNA3.1 (control) by neomycin selection with G418, using constructs we have described previously (9). HeLa cells were purchased from ATCC. All cell lines were maintained in Dulbecco’s modified eagle medium containing glucose (4.5 g/L) (Life Technologies) and supplemented with 10% fetal calf serum (FCS, SeraLabs), penicillin (100 IU/mL), streptomycin (100 µg/mL), and glutamine (6 mM), all purchased from Life Technologies. Cell lines used were authenticated and routinely confirmed to be negative for any *Mycoplasma* contamination. Hypoxia was achieved by incubating cells in 1% O_2_, 5% CO_2_, and 94% N_2_ in a Ruskinn SCI-tive workstation, without agitation.

### Antibodies and Reagents

The rabbit polyclonal CHCHD4 and rabbit polyclonal BNIP3 antibodies were purchased from Cambridge Biosciences. The mouse monoclonal HIF-1α antibody was purchased from BD Biosciences. The mouse monoclonal β-actin and rabbit polyclonal ATP5B antibodies were purchased from Abcam. The mouse monoclonal anti-myc (9B11) and rabbit monoclonal COXIV (3E11) antibodies were purchased from Cell Signaling Technology. The mouse monoclonal anti-pimonidazole antibody was purchased from HypoxyProbe, Inc. The donkey anti-rabbit and anti-mouse horseradish peroxidase-linked secondary antibodies were purchased from VWR. Sodium azide was purchased from Sigma-Aldrich and used at 5 mM for 16 h in order to optimally block HIF-1α protein levels without significantly affecting cell viability. DAPI solution (10 mg/mL) was purchased from Cambridge Biosciences. Pimonidazole (200 mM) was purchased from HypoxyProbe, Inc.

### Immunofluorescent Microscopy

Cells were seeded onto 13 mm ø coverslips, and after treatment, were fixed in either 4% paraformaldehyde (PFA) for 15 min (for assessment of intracellular hypoxia by pimonidazole staining) or 100% ice-cold methanol overnight (for assessment of the mitochondrial distribution by COXIV or ATP5B). Notably, we found that COXIV and ATP5B total protein levels in cells were not significantly affected by either prolonged exposure to hypoxia (1% O_2_) or stable increased expression of CHCHD4 and as such, we deemed these mitochondrial proteins as suitable markers for immunostaining mitochondria in the experiments described in this study. For PFA fixed samples, cells were washed and permeabilized with a 0.5% Triton-X solution for 10 min, then washed with phosphate-buffered saline. Immunostaining was carried out using primary antibodies followed by fluorescently labeled secondary antibodies (anti-mouse Alexa 568 and anti-rabbit Alexa 488, Life Technologies) as well as DAPI. All cell imaging was carried out using a DMI4000 B inverted microscope (Leica).

### Mitochondrial Distribution Analysis

Mitochondrial distribution analyses was carried out using Cell Profiler Image analysis software (27), and a custom image analysis pipeline was applied to each data set, as follows: images were loaded, and blue, green, and red channels were split into separate greyscale images and named “nuclei,” “mitochondria,” and “cell,” respectively. Individual, whole DAPI-stained (blue) fluorescent nuclei were identified as objects between 50 and 450 pixel units in diameter. Cell boundaries were identified using anti-α-tubulin (red) fluorescence, with Ridler–Calvard thresholding adopted to distinguish between cell edges. Only images of whole, entire nuclei, and/or whole entire cells were included. Nuclei or cells with their boundaries touching the edge of the image were excluded. Nuclear (blue) fluorescence was subtracted from the associated cell fluorescence with the resulting fluorescent shape identified as the cytoplasmic compartment. Each cytoplasmic compartment was divided into five equally spaced concentric rings (to control for different cell size), starting from the boundary of the nucleus. The mitochondrial (green) fluorescence intensity was measured in each concentric ring and provided as a fraction of the total cytoplasmic compartment fluorescence per cell. The sum of the two concentric areas closest to the nuclei was taken as the perinuclear region, while the two outermost concentric areas were taken as the peripheral region. At least 10 fields of view were analyzed per condition, per experiment, and all data points expressed with mean indicated (solid black bar).

### Electron Microscopy

Cells were seeded onto glass coverslips (ø 13 mm, VWR) and fixed in 2% PFA and 1.5% glutaraldehyde in 0.1 M cacodylate buffer. Samples were post-fixed in 1% OsO_4_ (osmium tetraoxide) and 1.5% potassium ferrocyanide [K_4_Fe (CN)_6_] in 0.1 M cacodylate buffer. Samples were dehydrated using a graded ethanol–water series, cleared in propylene oxide, and infiltrated with agar-100 resin. Ultrathin sections were cut and collected on 300-mesh grids and stained with lead citrate. Sections were viewed using a Joel 1010 transition electron microscope and images were recorded using a Gatan Orius CDD camera. Mitochondrial section diameter was determined relative to the scale bar, and mitochondria grouped above and below 1 µm diameter. At least 20 fields of view were analyzed per condition.

### Gene Silencing

Non-silencing siRNA duplexes (universal negative control #1) and custom designed siRNA duplexes were purchased from Sigma-Aldrich and transfected into sub-confluent cells using HiPerfect transfection reagent (QIAGEN) according to the manufacturer’s instructions. The target sequences for *CHCHD4* were 5′-GAGG AAACGTTGTGAATTA-3′ (siRNA(1)) and 5′-AAGATTTGGAC CCTTCCATTC-3′ (siRNA(2)). The target sequences for *HIF1A* were 5′-TACGTTGTGAGTGGTATTATT-3′ (siRNA(1)) and 5′-TAGA AGGTATGTGGCATTTAT-3′ (siRNA(2)).

### Gene Expression Analysis

Total RNA samples were isolated using the GeneElute kit and protocol (Sigma-Aldrich). cDNA synthesis was carried out using the qScript synthesis kit and protocol (Quantabio). mRNA expression was measured by quantitative (Q)PCR using SYBR Green Mastermix (Eurogentec Ltd.) and the DNA Engine Opticon 2 system (BioRad). The QPCR primer sequences were as follows: *CHCHD4_*F 5′-GAGCTGAGGAAGGGAAGGAT-3′, *CHCHD4_*R 5′-AATCCATGCTCCTCGTATGG-3′; *HIF1A_*F 5′-TCCAAGAAGCCCTAACGTGT-3′, *HIF1A_*R 5′-TGATCGTCTGGCTGCTGTAA-3′; *CA9_*F5′-GCCGCCTTTCTGGAGGA-3′, *CA9_*R 5′-TCTTCCAAGCGAGACAGCAA-3′; *BNIP3_*F 5′-GATATGGGATTGGTCAAGTCG-3′, *BNIP3_*R 5′-CGCTCGTGTTCCTCATGCT; *ACTB_*F 5′-CCCAGAGCAAGAGAGG-R′, *ACTB_*R 5′-GTCCAGACGCAGGATG.

### Statistics

Values are expressed as mean ± SD unless otherwise stated and statistical significance was determined by a 2-tailed unpaired Student’s *t-*test, with significance set at *p* < 0.05.

## Discussion

Mitochondria are organelles that exist in dynamic interrelated forms within a cellular mitochondrial network (28). It is now becoming clear that as such, mitochondria can influence many cellular processes and communicate with other organelles and intracellular machinery through changes in mitochondrial dynamics (fission and fusion), subcellular localization and morphology (29). As the major sites of oxygen consumption within the cell, precisely how changes in intracellular oxygenation affect mitochondrial behavior is of particular interest since hypoxia underlies the pathology of a broad range of diseases including cancer.

Previous studies in various cell contexts have shown that hypoxia induces a redistribution of mitochondria to the perinuclear region of the cell (8, 15, 20, 30) and regulates mitochondrial dynamics and morphology, promoting enlarged, donut-like (also known as toroid) mitochondria (7, 20, 31). Here, we have demonstrated that hypoxia stimulates perinuclear accumulation of mitochondria in a CHCHD4-dependent manner.

We identified human CHCHD4 previously using a functional genomics approach and showed that it is crucial for regulating hypoxic (HIF) responses, basal OCR and metabolism in tumor cells (9). CHCHD4 is involved in importing and folding CHCH-domain-containing mitochondrial proteins as part of the DRS within the IMS (12) and in doing so, links the DRS to respiratory chain function. Interestingly, mutations in the sulfhydryl oxidase GFER (which recycles CHCHD4 within the DRS) are associated with changes in mitochondrial morphology *via* changes in the mitochondrial recruitment of the fission protein DRP-1 (18).

We discovered previously that increased expression of *CHCHD4* in human cancers (e.g., breast, glioma, and pancreatic) is associated with the upregulation of hypoxia regulated genes and poor patient survival (9). Here, we found that increased expression of CHCHD4 in tumor cells resulted in perinuclear accumulation of the mitochondria, intracellular hypoxia associated with the perinuclear region and constitutive HIF activation in normoxia. Hypoxia within the tumor microenvironment is a well-known driver of tumor cell invasiveness and resistance to therapy (32), mediated primarily through the upregulation of HIF targets that enable tumor cells to survive and metabolically adapt (25, 26). Mitochondria are uniquely placed to influence intracellular oxygenation and communicate to HIF. Indeed, the ability for mitochondria to localize within close proximity to the nucleus in response to hypoxia has been proposed to enable mitochondrial ROS to accumulate within the nucleus and enhance the transcription of genes such as *VEGF* through promoter oxidation (8). Our study describes for the first time that regional (perinuclear) intracellular hypoxia is associated with increased basal transcription of HIF target genes.

Interestingly, we found that perinuclear mitochondria in CHCHD4 (WT)-expressing cells could be redistributed toward the periphery of the cell by transient (siRNA) depletion of HIF-1α or by treatment with the complex IV inhibitor, sodium azide. Notably, we found that the intracellular hypoxia observed in CHCHD4 (WT)-expressing cells was reduced by sodium azide treatment, while depletion of HIF-1α had no significant effect (data not shown). Based on our findings we propose that CHCHD4’s upstream control of respiratory chain function and basal OCR (at complex IV) is important for regulating intracellular oxygenation and HIF-1α signaling, while perinuclear localization of mitochondria is downstream.

As well as HIF-1α, HIF-2α protein is also dynamically induced over time in hypoxia, and it will be important to establish what role, if any, HIF-2α might play in regulating mitochondrial distribution and morphology. Furthermore, the response to hypoxia includes HIF-dependent metabolic adaptations which involve substrate switching and the reprogramming of metabolic pathways to maintain energetic and biosynthetic homeostasis (33). Thus, the prolonged time-frame for perinuclear accumulation of the mitochondria to occur in hypoxia (72 h) may reflect the metabolic and bioenergetic consequences of HIF activation. For example, in hypoxia, glucose consumption is elevated to support anaerobic ATP generation, while glutamine is diverted from the TCA cycle, and undergoes reductive carboxylation to support the production of lipids for membrane biosynthesis, both of which depend on HIF-1 signaling. Extended culture of cells in hypoxia is likely therefore to lead to the depletion of different metabolic substrates from, and accumulation of different metabolic by-products (e.g., lactate) in the culture medium compared to cells cultured in normoxia. It has been shown that substrate starvation has significant effects on mitochondrial motility (34) and morphology (35), and these two parameters are known to be intimately linked to the bioenergetic status of the mitochondria. Therefore, it will be of interest to investigate whether metabolic substrate availability (increased or decreased) is able to affect perinuclear accumulation of the mitochondria in response to changes in intracellular oxygenation.

Understanding the molecular mechanisms underlying how tumors respond to hypoxia is of great importance both in devising prognostic tests and for identifying improved therapeutic strategies for cancer treatment. Indeed, recent studies have highlighted advances in imaging mitochondrial dynamics associated with hypoxia in tumors for potential use in diagnosis (36) or as a biomarker for response to therapy (37). Further work exploring CHCHD4’s role in regulating mitochondrial dynamics should reveal how CHCHD4 or specific CHCHD4 substrates control intracellular oxygenation, HIF signaling and the distribution of the mitochondrial network.

## Ethics Statement

This study did not involve human participation, personal data, or use of human tissue.

## Author Contributions

LT designed and performed experiments, analyzed data, and contributed to writing the manuscript. OS generated stable CHCHD4 cell lines. MT performed electron microscopy analyses. MA provided the concept for the study, designed experiments, analyzed data, and wrote the manuscript.

## Conflict of Interest Statement

The authors declare that the research was conducted in the absence of any commercial or financial relationships that could be construed as a potential conflict of interest.
