# Corrigendum: CHCHD4 Regulates Intracellular Oxygenation and Perinuclear Distribution of Mitochondria

**DOI:** 10.3389/fonc.2019.00023

**Published:** 2019-01-23

**Authors:** Luke W. Thomas, Oliver Staples, Mark Turmaine, Margaret Ashcroft

**Affiliations:** ^1^Department of Medicine, University of Cambridge, Cambridge Biomedical Campus, Cambridge, United Kingdom; ^2^Centre for Cell Signalling and Molecular Genetics, Division of Medicine, University College London, London, United Kingdom; ^3^Department of Cell and Developmental Biology, University College London, London, United Kingdom

**Keywords:** hypoxia, hypoxia-inducible factor-1α, mitochondria, mitochondrial localization, perinuclear, coiled-coil helix coiled-coil helix domain containing 4

In the original article, there was an error. The name of parental cell line and antibiotic selection used should read “(U2OS)” and “neomycin selection with G418” respectively.

A correction has been made to the **Materials and Methods**, **Cell Culture**, paragraph one:

“Human U2OS osteosarcoma (U2OS-HRE-luc) cells have been described by us previously (17) and stably express a luciferase reporter construct under the control of a hypoxia response element, allowing us to monitor HIF/HRE activity alongside measuring mitochondrial endpoints. U2OS osteosarcoma (U2OS) cells were used to generate stable independent clonal cell lines (WT.cl1 and WT.cl3)-expressing CHCHD4.1 cDNA (CHCHD4 (WT)-expressing cells), CHCHD4-C66A/C668A cDNA (CHCHD4 (C66A/C68A)-expressing cells) or pcDNA3.1 (control) by neomycin selection with G418, using constructs we have described previously (9). HeLa cells were purchased from ATCC. All cell lines were maintained in Dulbecco's modified eagle medium containing glucose (4.5 g/L) (Life Technologies) and supplemented with 10% fetal calf serum (FCS, SeraLabs), penicillin (100 IU/mL), streptomycin (100 μg/mL), and glutamine (6 mM), all purchased from Life Technologies. Cell lines used were authenticated and routinely confirmed to be negative for any *Mycoplasma* contamination. Hypoxia was achieved by incubating cells in 1% O_2_, 5% CO_2_, and 94% N_2_ in a Ruskinn SCI-tive workstation, without agitation.”

The authors apologize for this error and state that this does not change the scientific conclusions of the article in any way. The original article has been updated.

